# Cost-related underuse of medications in older adults: ELSI-Brazil

**DOI:** 10.11606/S1518-8787.2018052000622

**Published:** 2018-10-25

**Authors:** Antônio Ignácio de Loyola, Josélia Oliveira Araújo Firmo, Juliana Vaz de Melo Mambrini, Sérgio Viana Peixoto, Paulo Roberto Borges de Souza, Fabíola Bof de Andrade, Maria Fernanda Lima-Costa, Francisco de Assis Acúrcio

**Affiliations:** I Fundação Oswaldo Cruz. Instituto René Rachou. Núcleo de Estudos em Saúde Pública e Envelhecimento. Belo Horizonte, MG, Brasil; II Fundação Oswaldo Cruz. Instituto René Rachou. Programa de Pós-Graduação em Saúde Coletiva. Belo Horizonte, MG, Brasil; III Universidade Federal de Minas Gerais. Escola de Enfermagem. Departamento de Enfermagem Aplicada. Belo Horizonte, MG, Brasil; IV Fundação Oswaldo Cruz. Instituto de Comunicação e Informação Científica e Tecnológica em Saúde. Rio de Janeiro, RJ, Brasil; V Universidade Federal de Minas Gerais. Faculdade de Farmácia. Departamento de Farmácia Social. Belo Horizonte, MG, Brasil

**Keywords:** Underuse of Medications, Prescription Drugs, Health of the Older Adults, Cross-Sectional Study, Pharmacoepidemiology, Subutilização de Medicamentos, Medicamentos Prescritos, Saúde do Idoso, Estudo Transversal, Farmacoepidemiologia

## Abstract

**OBJECTIVE:**

To assess the prevalence and factors associated with cost-related underuse of medications in a nationally representative sample of Brazilians aged 50 years and over.

**METHODS:**

Among the 9,412 participants of the Brazilian Longitudinal Study of Aging (ELSI-Brazil), 6,014 reported using at least one medication on regular basis and were included in the analysis. Underuse of medications was by stopping taking or reducing the number of tablets or the dose of any prescribed medication for financial reasons. The theoretical framework used for the selection of the exploratory variables included predisposing factors, enabling factors, and factors of need. Associations were tested by Poisson regression.

**RESULTS:**

The prevalence of underuse of medications was 10.6%. After adjustments for relevant covariables, positive and statistically significant associations (p < 0.05) with the outcome were found for females [prevalence ratio (PR) = 1.39], sufficiency of the family income for expenses (PR = 1.74 for sometimes and PR 2.42 for never), frequency with which the physician explains about the disease and treatment (PR = 1.31 for rarely or never), number of medications used (PR = 1.39 for 2–4 and 1.53 for 5 or more), fair (PR = 2.02) and poor or very poor self-rated health (PR = 2.92), and a previous medical diagnosis of depression (PR = 1.69). Negative associations were observed for the age groups of 60–79 years (PR = 0.75) and 80 years and over (PR = 0.43), socioeconomic status of the household (PR = 0.70, 0.79, and 0.60 for the second, third, and fourth quartile, respectively), and private health plan coverage (PR = 0.79). There were no associations between hypertension and self-reported diabetes and underuse of medications.

**CONCLUSIONS:**

Cost-related underuse of medications is multidimensional and complex, and it covers socio-demographic characteristics, health conditions, and the use of health services. The explanation about the disease and its treatment to the patient and the expansion of the universal access to pharmaceutical care can minimize the risks of underuse.

## RESUMO

**OBJETIVO:**

Determinar a prevalência e os fatores associados à subutilização de medicamentos por motivos financeiros em amostra nacional representativa da população brasileira com 50 anos ou mais.

**MÉTODOS:**

Entre 9.412 participantes do Estudo Longitudinal sobre a Saúde dos Idosos Brasileiros (ELSI-Brasil), 6.014 informaram usar medicamento(s) de uso contínuo e foram incluídos na análise. A subutilização de medicamentos foi definida como ter, por motivos financeiros, deixado de tomar ou ter diminuído o número de comprimidos ou a dose de algum medicamento receitado pelo médico. O marco teórico empregado para a seleção das variáveis exploratórias incluiu fatores predisponentes, capacitantes e de necessidade. As associações foram testadas por meio de regressão de Poisson.

**RESULTADOS:**

A prevalência de subutilização de medicamentos foi de 10,6%. Após ajustes pertinentes, associações positivas e estatisticamente significantes (p < 0,05) com o desfecho foram observadas para o sexo feminino [razão de prevalência (RP) = 1,39]; renda familiar às vezes (RP = 1,74) e nunca (RP = 2,94) suficiente para as despesas; frequência com que o médico explica sobre a doença e tratamento (RP = 1,31 para raramente ou nunca); número de medicamentos utilizados (RP = 1,39 para 2–4 e 1,53 para 5 ou mais); autoavaliação da saúde razoável (RP = 2,02) e ruim ou muito ruim (RP = 2,92); e diagnóstico médico de depressão (RP = 1,69). Associações negativas foram observadas para idade igual a 60–79 (RP = 0,75) e 80 anos ou mais (RP = 0,43), posição socioeconômica do domicílio (RP = 0,70; 0,79 e 0,60 para o segundo, terceiro e quartil superior) e cobertura por plano privado de saúde (RP = 0,79). Não foram observadas associações entre hipertensão e diabetes autorreferidos e subutilização de medicamentos.

**CONCLUSÕES:**

A subutilização de medicamentos por motivos financeiros tem caráter multidimensional e complexo, abrangendo características sociodemográficas, de condições de saúde e de utilização de serviços de saúde. Esclarecer ao paciente sobre a doença e o seu tratamento, e ampliar o acesso universal à assistência farmacêutica, podem minimizar os riscos da subutilização.

## INTRODUCTION

Medication use increases with age in terms of frequency (prevalence of use in the population) and intensity (amount of medications used)^1^. This is due to the greater presence of diseases and chronic conditions, whose treatment has an important support in pharmacotherapy^2^. This greater use results in a complex therapeutic regimen, intensifies adverse drug reactions (especially in aged organisms), and increases costs^3^, which may lead to non-adherence to prescribed treatment.

Underuse of medications is a type of non-adherence to drug treatment. There are different types of underuse, such as not acquiring all prescribed medications, reducing the number of tablets, or changing the dose used^4^. Underuse may be the last obstacle to the access to an effective therapy, when other barriers (inaccessibility to service and inadequate prescriptions) are overcome. It compromises the effectiveness of treatment, which increases health risks and, consequently, overload health services^5^. The increase in the price of medications, the different mechanisms that cover their costs (public or private), and the user’s purchasing power are the main reasons for cost-related underuse of medications, but they are not their only determinants^4^.

International studies have reported that, in addition to contextual financial pressures and the organization of health services, other factors influence the underuse of medications, such as sociodemographic characteristics (sex, age, education level) and health conditions (self-rated health and chronic diseases)^6–11^.

In Brazil, two studies have investigated cost-related underuse of medications among older persons living in the Metropolitan Region of Belo Horizonte^12^ ^,^ ^13^, but no previous study has investigated this issue in a nationally based sample of older Brazilians. Thus, this study aimed to investigate the prevalence and individual factors associated with cost-related underuse of medications in a nationally representative sample of the Brazilian population aged 50 years and over.

## METHODS

### Study population

This cross-sectional study used baseline data from the Brazilian Longitudinal Study of Aging (ELSI-Brazil), which is a nationwide population-based study on the living, health, and well-being conditions of the Brazilian population aged 50 years and over. The baseline survey was conducted in 2015 and 2016. The sampling design was based on selection stages, which combined municipality, census tract, and household. Municipalities were allocated in four strata according to their population size: (1) ≤ 26,700 inhabitants, (2) 26,701–135,000 inhabitants, (3) 135,001–750,000 inhabitants, and (4) > 750000 inhabitants. In the first three strata, sampling was carried out in three stages (municipalities, census tract, and household); in the fourth stratum, all municipalities were selected and the sampling was carried out in two stages (census tract and household). All persons aged 50 years and over living in the sampled households were considered eligible for the study. Sample size was defined as 10,000 persons (9,412 participated). For our investigation, we selected the participants who reported using one or more medications prescribed by a physician on a regular basis (n = 6,647). This and other data for this analysis were obtained with face-to-face interviews conducted at the household. More details on the ELSI-Brazil can be seen in another publication^14^.

### Variables and Data Collection

The outcome variable was cost-related underuse of medications, measured by the question: “In the last 30 days, because of financial problems, did you: (a) stopped taking, (b) decreased the number of, (c) decreased the dose of (dividing the tablet or taking fewer doses) any medication prescribed by a physician?”. We considered underuse the positive answer to any of the alternatives.

Exposure variables were selected based on the behavioral model of access and use of health services of Andersen^15^. This model lists the predisposing factors (sociodemographic characteristics), enabling factors (community and individual resources that favor or hinder access to services), and factors of need (health conditions) to explain the event. Although this theoretical framework was initially proposed to analyze the use of health services, the model has supported studies on the use of medications^2^ ^,^ ^7^ ^,^ ^10^ ^,^ ^13^. In this analysis, the predisposing factors included sex, age (50–59, 60–79, ^3^ 80 years), years of schooling (0, 1–10, 11 or more), marital status (married, single or separated, widow/widowed), and living arrangements (living alone or not). The enabling factors were region of residence, socioeconomic status of the household, sufficiency of the family income for expenses, health plan coverage, frequency with which the physician explains about the disease and the proposed treatment, and number of medications used. The socioeconomic position of the household was measured by a score created through principal component analysis^16^, which considered the number of household appliances and number of vehicles, in addition to the presence of domestic workers. Factors of need were self-rated health and history of medical diagnosis of hypertension and diabetes.

### Data Analysis

Initially, we examined the distribution of characteristics of the study participants according to the underuse of medications using Pearson’s chi-square test with Rao-Scott correction. The analyses of the associations were based on prevalence ratios and 95% confidence intervals, which were estimated by univariate and multivariate Poisson regression, with robust variance. The multivariate models included those variables that showed associations with the outcome at p < 0.20 level (Wald test) in the univariate analysis. The multivariate analysis started with the full model, followed by the selective deletion of variables according to the p value (backward), and successive models were compared by the likelihood-ratio test. The adequacy of the final model was evaluated by deviance statistics (p > 0.05). Those variables that showed an association with the outcome at p < 0.05 level were considered as independently associated with underuse of medication. Statistical analysis was conducted using the software Stata®, version 14.0, considering the sample weight of the individuals and the sample parameters (svy command).

The ELSI-Brazil was approved by the Research Ethics Committee of the *Instituto de Pesquisas René Rachou* of the *Fundação Oswaldo Cruz*, Minas Gerais (CAAE 34649814.3.0000.5091). All participants signed the informed consent.

## RESULTS

Of the 6,647 persons eligible for the study, 6,014 participated in this analysis. Exclusions were due to incomplete information of at least one of the variables considered. Excluded individuals did not differ from the participants in relation to sex and age (p > 0.05). Among participants, mean age was 64.3 years (SD = 10.0) and females were predominant (59.2%).

The prevalence of underuse of medications in the last 30 days was 10.6% (95%CI 9.3-12.1) and it was higher among women (12.5%; 95%CI 10.7–14.6) than among men (7.9%; 95%CI 6.8–8.2). This prevalence decreased progressively with age, in both sexes (Figure).

**Figure:**
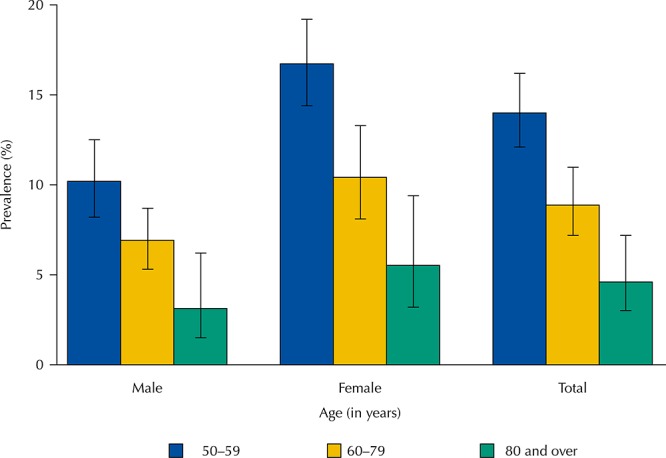
Prevalence of cost-related underuse of medications according to age group, for total population, and stratified by sex. Brazilian Longitudinal Study of Aging (ELSI-Brazil), 2015–2016.

Table 1 shows the results of the univariate analyses of the associations between predisposing factors and underuse of medications. There were statistically significant associations (p < 0.05) with the female sex, widowhood, and education level. No association was found with living alone (p = 0.084).

**Table 1:** Distribution of predisposing variables according to cost-related underuse of medications. Brazilian Longitudinal Study of Aging (ELSI-Brazil), 2015–2016. (n = 6,014)

Predisposing variables	Study population^a^	Underuse of medications^b (%)^	PR^c^	95%CI	p^d^
Sex					< 0.001
Male	2,278	7.9	1.00		
Female	3,736	12.5	1.58	1.31–1.91	
Age (years)					
50–59	2,133	14.0	1.00		< 0.001
60–79	3,284	8.9	0.64	0.53–0.77	
80 and over	597	4.6	0.33	0.22–0.51	
Education level (years)					0.012
Zero	949	12.3	1.00		
1–10	3,756	11.4	0.93	0.74–1.16	
11 or more	1,309	7.8	0.63	0.47–0.85	
Marital status					0.014
Married	3,443	10.6	1.00		
Single/Separated	1,278	12.9	1.21	0.99–1.41	
Widow/Widowed	1,293	8.2	0.78	0.61–0.99	
Living alone					0.084
No	5,241	10.8	1.00		
Yes	773	8.8	0.81	0.63–1.06	

^a^ Unweighted.

^b^ Weighted by sampling design

^c^ PR (95%CI): prevalence ratio (95% confidence interval), estimated by Poisson regression model with robust variance.

^d^ Pearson’s chi-square test, for comparison of proportions, with Rao-Scott correction.

All the enabling factors, except for geographical region (p = 0.278), showed statistically significant associations with underuse of medications (Table 2). They are: socioeconomic status of the household, sufficiency of income for expenses, private health plan coverage, explanation of the disease and treatment by the physician, and number of medical visits and medications used.

**Table 2:** Distribution of enabling variables according to cost-related underuse of medications. Brazilian Longitudinal Study of Aging (ELSI-Brazil), 2015–2016. (n = 6,014)

Enabling variables	Study population^a^	Underuse of medications^b (%)^	PR^c^	95%CI	p^d^
Geographic region					0.278
Southeast	2,697	10.2	1.00		
Northeast	1,501	12.2	1.20	0.97–1.47	
North	353	14.2	1.39	0.99–1.96	
South	851	9.2	0.90	0.68–1.20	
Midwest	612	10.4	1.02	0.75–1.39	
Socioeconomic status (in quartiles)					< 0.001
Q1	1,557	15.5	1.00		
Q2	1,550	11.1	0.72	0.57–0.90	
Q3	1,502	11.1	0.72	0.57–0.89	
Q4	1,405	5.5	0.36	0.27–0.48	
Sufficiency of family income for expenses					< 0.001
Always	2,037	4.0	1.00		
Sometimes	1,520	8.1	2.04	1.49–2.79	
Never	2,457	18.2	4.59	3.57–5.90	
Health insurance coverage					< 0.001
No	4,388	12.3	1.00		
Yes	1,626	6.3	0.51	0.41–0.64	
Physician explains about disease and treatment					< 0.001
Always	4,960	9.3	1.00		
Rarely/Never	1,054	16.9	1.81	1.49–2.21	
No. of medical appointments in the last 12 months					0.019
0–1	1,384	8.4	1.00		
2–3	1,992	10.8	1.29	1.00–1.66	
4 or more	2,638	11.7	1.40	1.10–1.78	
No. of medications used					< 0.001
1	1,527	6.9	1.00		
2–4	3,328	11.4	1.65	1.30–2.09	
5 or more	1,159	13.7	1.98	1.50–2.61	

^a^ Unweighted by sample weight.

^b^ Weighted by sample weight.

^c^ PR (95%CI): prevalence ratio (95% confidence interval), estimated by Poisson regression model with robust variance.

^d^ Pearson’s chi-square test with Rao-Scott correction.

Self-rated health and history of medical diagnosis of depression showed statistically significant associations with underuse of medications. The history of medical diagnosis of hypertension and diabetes was not found to be associated with the outcome [p = 0.293 and p = 0.623, respectively (Table 3)].

**Table 3:** Distribution of the variables of health needs according to cost-related underuse of medications. Brazilian Longitudinal Study of Aging (ELSI-Brazil), 2015–2016. (n = 6,014)

Predisposing variables	Study population^b^	Underuse of medications^c (%)^	PR^d^	95%CI	p^e^
Self-rated health					< 0.001
Very good/Good	2,299	4.3	1.00		
Fair	2,905	11.9	2.75	2.16–3.53	
Poor/Very poor	810	24.9	5.76	4.44–7.47	
Hypertension^a^					
No	1,880	11.7	1.00		
Yes	4,134	10.6	0.92	0.77–1.11	0.293
Diabetes^a^					
No	4,718	10.5	1.00		
Yes	1,296	11.1	1.05	0.86–1.30	0.623
Depression^a^					< 0.001
No	4,685	7.9	1.00		
Yes	1,329	19.9	2.51	2.11–2.98	

^a^ Previous medical diagnosis

^b^ Unweighted.

^c^ Weighted by sampling design .

^d^ PR (95%CI): prevalence ratio (95% confidence interval), estimated by Poisson regression model with robust variance.

^e^ Pearson’s chi-square test with Rao-Scott correction.

The final results of the multivariate analysis of the factors associated with underuse of medications are presented in Table 4. Among those predisposing factors, independent and statistically significant associations (p < 0.05) were found for female sex (PR = 1.39) and age (PR = 0.75 and 0.43 for the age group of 60–79 and 80 years and over, respectively, compared to younger persons). Regarding enabling factors, the socioeconomic status of the household (RP = 0.70, 0.79, and 0.60 for the second, third, and fourth quartiles (in relation to the first one), respectively) and private health insurance coverage (PR = 0.79) showed negative associations. Positive associations were found for sufficiency of family income for expenses (RP = 1.74 and 2.94 for sometimes and never, respectively, compared to always), frequency with which the physician explains about the disease and treatment (PR = 1, 31 for rarely or never), and number of medications used (PR = 1.39 for 2–4 and 1.53 for 5 or more). Among factors of need, poorer self-rated health (PR = 2.02 and 2.92 for fair and poor or very poor, respectively) and history of medical diagnosis of depression (PR = 1.69) showed positive associations.

**Table 4:** Final results of the multivariate analysis of the characteristics associated with cost-related underuse of medications. Brazilian Longitudinal Study of Aging (ELSI-Brazil), 2015–2016.

Variable	Adjusted PR^a^	95%CI
Sex (ref: male)		
Female	1.39	1.16–1.67^b^
Age (ref: 50–59 years)		
60–79	0.75	0.63–0.89^c^
80 and over	0.43	0.28–0.67^b^
Socioeconomic status in quartiles (ref: Q1)		
Q2	0.70	0.57–0.87^c^
Q3	0.79	0.64–0.97^c^
Q4	0.60	0.44–0.77^b^
Sufficiency of monthly family income for expenses (ref: always)		
Sometimes	1.74	1.28–2.35^b^
Never	2.94	2.29–3.79^b^
Health insurance coverage (ref: no)		
Yes	0.79	0.63–0.98^c^
Frequency with which the physician explains about the disease and treatment (ref: frequently)		
Rarely/Never	1.31	1.09–1.57^c^
Number of medications used (ref: 1)		
2–4	1.39	1.11–1.74^c^
5 or more	1.53	1.16–2.02^c^
Self-rated health (ref: very good/good)		
Fair	2.02	1.60–2.57^b^
Poor/Very poor	2.92	2.23–3.81^b^
Depression (ref: no)		
Yes	1.69	1.41–2.02^b^

^a^ Adjusted PR (95%CI): prevalence ratio estimated by Poisson regression model with robust variance, adjusted for all variables described in the table.

^b^ p < 0.001

^c^ p < 0.05

## DISCUSSION

The main results of this analysis were: (1) approximately one in 10 study participants reported cost-related underuse of medications; (2) age, sex, and poor socioeconomic status (assessed by the score of household goods and perceived sufficiency of income) were important factors associated with underuse of medications; (3) the source of care (private health plan coverage), the quality of care (frequency with which the physician explains about the disease and treatment), and the number of medications used were also associated with the outcome; (4) persons with poorer self-rated health and with depression were more likely to have a cost-related underuse of medications. On the other hand, there were no associations between hypertension and diabetes and underuse of medications.

The frequency with which participants had a cost-related underuse of medications (10.6%) was similar to that observed among Canadian adults aged 18 years and over (9.9%)^10^ and higher than that observed in the United States, in the same age group (5.1%)^7^. In the age group of 60 years and over, the cost-related underuse of medications observed in this analysis (8.3%; data not shown) was higher in relation to European older adults (3.6%)^6^ but lower than that observed in the metropolitan area of Belo Horizonte, Brazil, (12.9%)^13^ and in the United States (20%)^9^. In addition to methodological issues related to how the outcome was defined, specificities of local policies for pharmaceutical care and coverage of the costs of medications may explain the above mentioned differences in prevalence.

Regarding predisposing factors, the prevalence of underuse of medications was higher among women and lower in older ages, regardless of the enabling factors and factors of need. Women complain more intensely and frequently of health problems and use more medications than men, which result in higher costs with pharmacotherapy^1^ ^,^ ^2^. Higher spending with medications increases the financial pressure of women, who are more vulnerable than men in terms of income, socioeconomic status, and education level^6^ ^,^ ^9^. As for age, our results are in line with what has been reported in other populations^4,8–10^, showing that the cost-related underuse of medications decreases with age. With aging, health concern and vigilance increases, and non-adherence to treatment is perceived as harmful^17^.

The enabling variables are those that are more subject to modifications from public policies and the way health services are organized^15^. The financing of the costs with medications (partial or total subsidy to the prescribed medication, linked to specific care programs) can alleviate the financial pressures that hinder access^18^ ^,^ ^19^. The quality of the medical consultation (prescriptions that promote the rational use of medications, provision of explanations, and guidance to the patient), as well as pharmaceutical care services, can reduce the risks of non-adherence to the prescribed medication^20^. Income and socioeconomic status are the enabling factors most directly related to cost-related underuse, since they enable the individual to acquire medications. Our results are in line with other investigations that show consistent associations between higher income and lower underuse of medications^4^ ^,^ ^7^ ^,^ ^8^ ^,^ ^10^ ^,^ ^13^.

As with European older adults^6^, Brazilian older adults who perceive their family income as insufficient for expenses underuse more medications, regardless of other relevant factors including the score of household goods. Perceived sufficiency of household income for expenses is an important indicator of financial difficulties. Individuals with a similar income, but with a perception that their income is insufficient for expenses, usually react differently to the cost pressures involved in drug treatment, thus reducing the use in the face of a small increase in cost or adhering completely to the prescription, given the significant increase in the costs of medications^2^ ^,^ ^11^. Possibly other issues are considered in this process, such as the perception of the health risks involved or the value given to the medication in the treatment of the disease^17^. Our results support the hypothesis that expenses with medications can be disregarded when financial difficulties compel the individual to prioritize family expenses^2^ ^1^.

Individuals covered by private health plan tend to present fewer financial barriers to the use of health services, as these barriers act by increasing social inequalities in access to these services. This could explain the lower underuse of medications observed among participants with health plan coverage, which is consistent with results found in other countries^4^ ^,^ ^7^ ^,^ ^8^ ^,^ ^10^ and in a Brazilian metropolitan region^13^.

The results regarding the greater underuse of medications among those with poorer socioeconomic status and those not covered by health plan reinforce the importance of governmental initiatives that seek to improve access to medications. The Brazilian Unified Health System (SUS) includes pharmaceutical care as a component of comprehensive health care, and the Brazilian Popular Pharmacy Program ^22^ is an important initiative to ensure this right to the citizen. This program can contribute with better adherence to pharmacological treatment among persons with lower socioeconomic level, especially those who live in places where public pharmaceutical care is not sufficiently organized. Recent studies have shown that, among Brazilians, a little more than a third of persons with hypertension and more than half of the persons with diabetes obtained at least one of the medications used to control their respective diseases in the Brazilian Popular Pharmacy Program ^23^, and approximately 72% of the medications used in the treatment of hypertension were obtained from the SUS and 16% was provided by this program ^24^. Our results showed no association of hypertension and diabetes with cost-related underuse of medications, in contrast to that observed for self-rated health and depression. This lack of association can be explained by the policies mentioned above.

As observed among Brazilian older adults (≥ 60 years)^13^ and among adults (≥ 18 years) from higher income countries^8^, our study identified a greater underuse of medications among those who did not feel well informed by the physician about their disease and treatment. Patients are more willing to overcome difficulties in acquiring medications when they are heard about their values and concerns (including financial ones) and when they have answers about the disease and its treatment^5^ ^,^ ^8^. The judgment of patients regarding accessibility to medication can vary and it may be difficult for the health professional to distinguish between inability to afford and unwillingness to purchase the medication needed for treatment^10^. Health professionals should be aware that the quality of information, including answering the patients’ questions, can increase adherence and, consequently, intensify the effectiveness of treatment.

In our study, the number of medications used was associated with cost-related underuse. In addition to financial pressures, the amount of prescribed medications is considered as the main determinant of underuse, followed by individual characteristics (perceptions of the risks or benefits of the treatment) and the medication itself (possible side effect, complex administration)^18^ ^,^ ^21^. Health professionals can contribute minimizing underuse in these conditions when associating the quality of the prescription at an affordable cost. To this end, they can prescribe cheaper medications or increase the time needed for a prescription renewal, as well as give advice about the possibility of obtaining the medication in public pharmacies or in programs that provide subsidized medication.

Among the factors of need, poorer self-rated health and a previous medical diagnosis of depression were independently and positively associated with underuse of medications. The results related to self-rated health corroborate that observed in other populations^7^ ^,^ ^8^ ^,^ ^10^ ^,^ ^13^. Positive associations between cost-related underuse of medications and depression have been shown in another study^8^. In the United States, among those with chronic diseases, depression was associated with underuse of prescribed medications and those specific for the treatment of depression. Persons with depression may have impaired cognitive function and lower levels of energy and motivation, which may affect their willingness to spend on medications^8^. It is possible that the judgment of patients on the role of the medication in preventing the progression of the disease or the perception of a transitory nature of the depression may favor underuse among depressed individuals. Positive associations between poor health status and cost-related underuse of medication are worrying. Persons with poorer health conditions are at greater risk for the clinical consequences of non-adherence to the proposed therapeutic regimen^10^, as these consequences are minimized or ignored.

This study presents some limitations that hinder a more in-depth interpretation of its results. These limits refer to the impossibility of identifying the underused medication, which could indicate more clearly whether underuse is selective. Selectivity may reflect perceptions about the value of the proposed treatment and the importance of including the medication in the treatment. Aspects related to the provision and organization of health services, especially in relation to pharmaceutical care, can also contribute to this selectivity. On the other hand, the strength of the study arises from its comprehensiveness and the representativeness of the sample, which gives it originality. This is the first study to investigate cost-related underuse of medications which allows inference of its results to the Brazilian adult population aged 50 years and over.

In summary, our results point to the multidimensional and complex nature of the cost-related underuse of medications. Given the importance assumed by the medication in the treatment of different diseases, the costs they represent to patients, and the health risks of not adhering to the proposed pharmacotherapy, health professionals should strive to make treatment feasible. In this sense, the discussion with the patient about the disease and its treatment, the adequacy of the prescription to the patient’s ability to pay, and the provision of information that helps to overcome barriers to available medications should be offered. Regarding health planners, actions that enhance the universalization of pharmaceutical care, such as regular availability of medications in pharmacies of the SUS and the Brazilian Popular Pharmacy Program, can certainly contribute to reduce the underuse of medications and promote equality and equity in the access to them with positive impacts on the health of the population.

## References

[1] Bertoldi AD, Dal Pizzol TS, Ramos LR, Mengue SS, Luiza VL, Tavares NUL (2016). Perfil sociodemográfico dos usuários de medicamentos no Brasil: resultados da PNAUM 2014. Rev Saude Publica.

[2] Jung Y, Byeon J, Chung H (2015). Prescription drug use among adults with chronic conditions in South Korea: dual burden of health care needs and socioeconomic vulnerability. Asia Pac J Public Health.

[3] Rollason V, Vogt N (2003). Reduction of polypharmacy in the elderly: a systematic review of the role of the pharmacist. Drugs Aging.

[4] Briesacher BA, Gurwitz JH, Soumerai SB (2007). Patients at-risk for cost-related medication nonadherence: a review of the literature. J Gen Intern Med.

[5] Glasziou P, Straus S, Brownlee S, Trevena L, Dans L, Guyatt G (2017). Evidence for underuse of effective medical services around the world. Lancet.

[6] Stankuniene A, Stankunas M, Avery M, Lindert J, Mikalauskiene R, Melchiorre MG (2015). The prevalence of self-reported underuse of medications due to cost for the elderly: results from seven European urban communities. BMC Health Serv Res.

[7] Law MR, Cheng L, Dhalla IA, Heard D, Morgan SG (2012). The effect of cost on adherence to prescription medications in Canada. CMAJ.

[8] Kemp A, Roughead E, Preen D, Glover J, Semmens J (2010). Determinants of self-reported medicine underuse due to cost: a comparison of seven countries. J Health Serv Res Policy.

[9] Zivin K, Ratliff S, Heisler MM, Langa KM, Piette JD (2010). Factors influencing cost-related nonadherence to medication in older adults: a conceptually based approach. Value Health.

[10] Kennedy J, Morgan S (2006). A cross-national study of prescription nonadherence due to cost: data from the joint Canada-United States Survey of Health. Clin Ther.

[11] Piette JD, Heisler M, Wagner TH (2004). Cost-related medication underuse among chronically ill adults: the treatments people forgo, how often, and who is at risk. Am J Public Health.

[12] Luz TCB, Loyola AI, Lima-Costa MF (2011). Perceptions of social capital and cost-related non-adherence to medication among the elderly. Cad Saude Publica.

[13] Luz TCB, Loyola AI, Lima-Costa MF (2009). Estudo de base populacional da subutilização de medicamentos por motivos financeiros entre idosos na Região Metropolitana de Belo Horizonte, Minas Gerais, Brasil. Cad Saude Publica.

[14] Lima-Costa MF, Andrade FB, Souza PRB, Neri AL, Oliveira Duarte YA, Castro-Costa E (2018). The Brazilian Longitudinal Study of Aging (ELSI-Brazil): objectives and design. Am J Epidemiol.

[15] Andersen RM (1995). Revisiting the behavioral model and access to medical care: does it matter?. J Health Soc Behav.

[16] Ismail K (2008). Unravelling factor analysis. Evid Based Ment Health.

[17] Costa CMFN, Silveira MR, Acurcio FA, Guerra AA, Guibu IA, Costa KS (2017). Utilização de medicamento pelos usuários da atenção primária do Sistema Único de Saúde. Rev Saude Publica.

[18] Tavares NUL, Bertoldi AD, Mengue SS, Arrais PSD, Luiza VL, Oliveira MA (2016). Fatores associados à baixa adesão ao tratamento farmacológico de doenças crônicas no Brasil. Rev Saude Publica.

[19] Stopa SR, Malta DC, Monteiro CN, Szwarcwald CL, Goldbaum M, Cesar CLG (2017). Acesso e uso de serviços de saúde pela população brasileira, Pesquisa Nacional de Saúde 2013. Rev Saude Publica.

[20] Lima MG, Álvares J, Guerra AA, Costa EA, Guibu IA, Soeiro OM (2017). Indicadores relacionados ao uso racional de medicamentos e seus fatores associados. Rev Saude Publica.

[21] Piette JD, Heisler M, Wagner TH (2006). Medication characteristics beyond cost alone influence decisions to underuse pharmacotherapy in response to financial pressures. J Clin Epidemiol.

[22] Ministério da Saúde (BR), Conselho Nacional de Saúde (2004). Resolução Nº 338 de 6 de maio de 2004. Aprova a Política Nacional de Assistência Farmacêutica.

[23] Costa KS, Tavares NUL, Mengue SS, Pereira MA, Malta DC, Silva JB (2016). Obtenção de medicamentos para hipertensão e diabetes no Programa Farmácia Popular do Brasil: resultados da Pesquisa Nacional de Saúde, 2013. Epidemiol Serv Saude.

[24] Mengue SS, Bertoldi AD, Ramos LR, Farias MR, Oliveira MA, Tavares NUL (2016). Acesso e uso de medicamentos para hipertensão arterial no Brasil. Rev Saude Publica.

